# Trust-Based Access Control Model from Sociological Approach in Dynamic Online Social Network Environment

**DOI:** 10.1155/2014/936319

**Published:** 2014-10-13

**Authors:** Seungsoo Baek, Seungjoo Kim

**Affiliations:** ^1^Department of Electronic Information Science, Korea Military Academy, Gongreung-dong, Nowon-gu, Seoul 139-799, Republic of Korea; ^2^CIST (Center for Information Security Technologies), Korea University, Anam-dong, Seongbuk-gu, Seoul 136-713, Republic of Korea

## Abstract

There has been an explosive increase in the population of the OSN (online social network) in recent years. The OSN provides users with many opportunities to communicate among friends and family. Further, it facilitates developing new relationships with previously unknown people having similar beliefs or interests. However, the OSN can expose users to adverse effects such as privacy breaches, the disclosing of uncontrolled material, and the disseminating of false information. Traditional access control models such as MAC, DAC, and RBAC are applied to the OSN to address these problems. However, these models are not suitable for the dynamic OSN environment because user behavior in the OSN is unpredictable and static access control imposes a burden on the users to change the access control rules individually. We propose a dynamic trust-based access control for the OSN to address the problems of the traditional static access control. Moreover, we provide novel criteria to evaluate trust factors such as sociological approach and evaluate a method to calculate the dynamic trust values. The proposed method can monitor negative behavior and modify access permission levels dynamically to prevent the indiscriminate disclosure of information.

## 1. Introduction

There has been an explosive growth in the use of the OSN (online social network). The OSN has become an indispensable element in our life. It is user-centered and relationship-oriented and provides the ability to freely share information. We use these features to communicate with friends and family members. We are able to develop relationships with people we have never met and yet with whom we share common interests. Similarly, the OSN has facilitated the expansion of social relationships that overcome the limitation of time and space.

However, the expansion of relationships has many side effects including privacy exposure, indiscriminate information sharing, and the dissemination of false information. Therefore, it has become common for people to consider the reliability of the people who provide information rather than the information itself. People focus on trust in the information sharing process. In the OSN environment, many people store their data in their domain. In addition, they can read or write content into other user's domains. A data owner can request the following requirements to control other user's access. The owner may allow only trusted people to access his domain. The data requestor may require access permission from the owner before accessing the data. The access policies made by the data owner must be regulated. These requirements in the OSN are available in access control applications such as MAC (mandatory access control) [1], DAC (discrete access control) [2], and RBAC (role-based access control) [3]. These traditional access control models, however, have limitations when applied to OSN environments. Because the user's relationship in the OSN is continuously changing, the static rules in the traditional access controls are vulnerable when applied to the OSN. For example, a trusted data requestor, who has been given wide range access control from the owner, may change his motivation and become a malicious user posting abusive comments or disclosing the owner's private information. Therefore, it is important for the owner to monitor the requestor's behavior continuously and ensure that he remains trustworthy.

The key contributions of this paper are as follows. First, we identify the criteria to evaluate the trust factors based on a sociological approach. Mayer et al. [4] suggested that the trust consists of internal and external factors. More specifically, tendency, ability, sustainability, and relationships between the trustor and the trustee can be principal components of trust. Second, we propose and evaluate a method to calculate the dynamic trust values for a user. Malicious users are strategic as their interests and are to exploit an owner's content. The proposed method can monitor negative behavior and modify access levels accordingly. Moreover, the proposed architecture provides a technique to update trust levels and deter malicious user behaviors.

This paper is organized as follows.  Section 2 presents a brief introduction to the definition of trust and the limitations of the traditional access control models in the OSN.  Section 3 introduces a method to calculate the trust value.  Section 4 describes the application of the dynamic trust value to the user's access level.  Section 5 presents the experimental results of several scenarios.  Section 6 concludes this paper.

## 2. Related Works

### 2.1. Definition of Trust

Trust is widely accepted as a major component of human social relationships. Based on trust, we choose a person with whom to communicate or decide, in fact, whether to communicate with a person. Many studies on trust have been presented. In psychology, trust is considered as a psychological state of a trustor, where his risks are vulnerable to the positive expectations of the trustee's behavior [5]. In sociology, Lik [6] defined trust as “the subjective expectation an entity has about another's future behavior.” Trust is not confirmed in a moment. It is the special value accumulated based on one's previous behavior or experience. It reduces uncertainty and risk of a future relationship between a trustor and a trustee.

The major entities of trust are classified as a trustor and trustee. A trustor evaluates the amount how much he credits a trustee. If the trustor already knows the trustee before evaluation, he can easily measure the value. However, it is evident that there is significant communication and information sharing among unknown people in the OSN environment. Thus, to evaluate the trust value correctly, several mechanisms have been studied. The representative examples use delegation of trust [7] and recommendation systems [8]. The former implies that people trust a friend of a friend. The latter requires that a trustor obtains indirect references such as a word-of-mouth recommendation from third parties who have experiences with the trustee in the past. Therefore, measuring trust values is considered a combination of the trustor's direct trust values and third parties' indirect trust values.

### 2.2. Threats and Security Requirements in OSN

As OSNs have developed into critical online communication platforms integrated into people's daily lives, the security risks have equally evolved. Malicious users or collectives fabricate the recommendation values spurious compliments and biased references. In a Sybil attack [9], an individual entity masquerades as multiple simultaneous identities. For example, adversaries can promote popularity and reputation of an account in e-commerce settings by voting the target account as “Good.” A malicious pretrusted peer can turn against a trustor after establishing a relationship and affect the trustor's trust value in a recommendation system [10]. Threats in the OSN related to trust are shown in Table 1.

### 2.3. Limitations of Traditional Access Control Mechanisms in OSN

Many OSN providers such as Facebook, Google+, and Linked-In have utilized access control mechanisms to manage the information flow and to prevent privacy breaches. From a relationship aspect, an individual user classifies his relationships into friends, families, and colleagues. From a data viewpoint, sensitive data can be grouped into public, private, or customized. These access control mechanisms are based on traditional static access control models such as MAC [1], DAC [2], and RBAC [3]. In addition to the traditional access control models, many studies on access control in the OSN have been conducted. Carminati et al. [11, 12] proposed a rule-based access control model that regulates the access information with relationship type, relationship depth, and trust value. Specifically, each trust value assigned to a person is static and subjective. However, these trust criteria have difficulties when applied to the current OSN environment because user relationships are persistently changing and the user trust is not fixed in the OSN. Moreover, they adapt the concept of trust transference, which implies that friends of a friend are reliable. For example, Alice trusts Bob as 0.5 and Bob, who is a friend of Alice, trusts Eve as 0.3. Therefore, the result of trust between Alice and Eve is 0.15. This is derived from the product of Alice's trust against Bob and Bob's trust against Eve. However, in the real world, each individual does not measure trust in the same manner. Trust value should be verified using a standard comparative method similar to that described in Section 3.

Hu and Ahn [13] and Yuan et al. [14] proposed an approach to multiparty and user-to-user relationship access control in the OSN. A user can have a relative multirole including owner, reader, contributor, and disseminator of contents. These roles have a designated policy calculated using their weight values. The consequence of the calculation influences the decision to permit access to the data. However, these advantages are to resolve policy conflicts between users. A user must set up all role's policies to share the data before granting permissions. This can be cumbersome when policies are changed.

The application of traditional and static access control mechanisms as described above has several disadvantages. First, it is difficult to change access policies before a user fixes his access permission. Second, the access control mechanism based on the relationships is not practical to apply to the dynamic environment of the OSN because the relationships change frequently. Third, a pretrusted user can become a malicious user. Thus, it is necessary to apply a dynamic adaptive access control mechanism in the OSN.  Table 2 compares the proposed dynamic trust-based access control model with previous access control models in the OSN.

## 3. Method for Evaluating Dynamic Trust

### 3.1. Sociological Trust Factors

Communication in the OSN is different from offline communication in that there is no physical contact with friends or colleagues and there exists only a limited knowledge of the anonymous characteristics. However, trials for establishing trust can lead to the development of relationships among people, even though they do not know each other directly. The value of trust is the degree that a subject will perform as expected in a certain context. The proposed model utilizes the concept of trust by assessing the trustee's previous behavior to predict his future behavior. Interactions and sustainable relationships enable a user to establish an expectation of future behavior. Those interactions imply more trust.

We classified entities for a trustor, trustee, and third party who provide indirect trust. A trustor is a subjective entity who must assess and decide whether an access requestor is permitted to access his data. A trustee must introduce himself to receive access permission from the trustor. The third party is an entity that provides indirect trust from previous experience with the trustee.

What are the requirements for establishing trust? Mayer et al. [4] say “Trust is the willingness of a party to be vulnerable to the actions of another party based on the expectation that the other will perform a particular action important to the trustor, irrespective of the ability to monitor or control that other party.” They identify trust factors as trustor's tendency, application, trustee's sustainability, and relationships between a trustor and trustee. We feel this definition applies to OSN services because these criteria can be mapped into current OSN services. The proposed trust evaluation factors and OSN services mapped by the criteria are shown in Table 3.

### 3.2. Calculating Dynamic Trust Value

Trust can change in the OSN environment. Adversaries such as malicious users or pretrusted users mentioned in Section 2 may change their motivation and become malicious users in a future transaction with the owner. However, it is difficult for the owner to recognize the change of a requester's intention to access information. Therefore, the trust value must evolve based on transactions and a continuous observation of the trustee's behavior.

#### 3.2.1. Direct Trust (DT)

A trustor is satisfied when a trustee accesses and uses the trustor's data as anticipated. That is, the trust of the trustor is the trustor's (*P*) feeling based on the trustee's (*Q*) positive behaviors. We define this trust as a trustor's direct trust (DT) against the trustee because trust is subjective based on direct experience in trustee's behavior. We adopt the definition of trust as proposed by Mayer et al. [4]. We calculate the current direct trust based on the factors in Table 3 according to trustor's tendency of trust criteria (ta, ts, tr⁡), ability (*A*), trustee's sustainability (*S*), and trustee's relationship (*R*). Current direct trust is calculated as

(1) $$\begin{array}{c} {\text{DT}}_{\text{current}}\left(P,Q\right)=\left(\text{ta}\times A\right)+\left(\text{ts}\times S\right)+\left(\mathrm{tr}\times R\right), \end{array}$$

where 0 < ta, ts, tr⁡≤1, and ta + ts + tr⁡ = 1.

*A*, *S*, and *R* matched the OSN services shown in Table 3. Hence, the level of direct trust is represented by the value DT_current_ (*P*, *Q*), where 0 ≤ DT_current_ (*P*, *Q*) ≤ 1, with zero referring to the trustor's trust against trustee as being totally untrusted and one for fully trusted. Trust changes with transaction (*t*) and trustee's behavior. Thus, the direct trust at the *t*th transaction is calculated as

(2) $$\begin{array}{c} {\text{DT}}_{t}\left(P,Q\right)=\left(1-\alpha \right)\times {\text{DT}}_{\text{current}}\left(P,Q\right)+\alpha \times {\text{DT}}_{t-1}\left(P,Q\right), \end{array}$$

where *α* is the influence rate of the previous direct trust and *t* − 1 is the past transaction.

#### 3.2.2. Indirect Trust (IT)

Direct trust is provided by past transactions. If users are unknown, a trust relationship can be established by assigning a default value. Assume that two users, unknown to each other, are willing to interact. It is difficult for the data owner to share his information with the unknown user. In a general manner, the data owner inquires about the requestor from his friends. For example, Alice and Bob are friends. Bob and Charlie are friends. However, Alice and Charlie do not know each other. Alice has similar characteristics to Bob. If Bob trusts Charlie, Alice may trust Charlie and can share her information with him. We call this a recommendation [15] and define it as indirect trust (IT). Indirect trust is the average direct trust value of third parties who have previous experiences with the trustee.

Beginning calculating the indirect trust, it is necessary for the trustor to understand the similarity of the other trustor against the trustee. People feel more reliable when they meet other people who share similar characteristics. Moreover, similar characteristics affect friendship and people who have more common friends feel closer to each other. Similarity is the degree of people having a likeness in a manner to assess trust. Thus, we assess similarity based on common friends and their direct trust that is derived from formula (2). The average distance (Dist) between the trustor and trustee is calculated as

(3) $$\begin{array}{c} \text{Dis}{\text{t}}_{t}\left(P,Q\right)=\frac{1}{N}\underset{x\in N}{\sum }\frac{1}{\text{D}{\text{T}}_{t}\left(P,x\right)\times \text{D}{\text{T}}_{t}\left(x,Q\right)}, \end{array}$$

where *N* is the number of common friends (*x*) between the trustor (*P*) and trustee (*Q*). We use a BFS (breadth first search) algorithm to determine common friends with the shortest paths [16]. The average distance reflects an increase or decrease in similarity. Similarity (Sim) increases when the average satisfaction distance is less than the distance threshold (*r*) and decreases when it is more than the distance threshold. Das and Islam [17] applied the concept of reward and punishment for calculating of similarity. We utilize reward (*τ*) and punishment (*ρ*) coefficients to update the similarity. We assigned a larger punishment coefficient because it is more difficult to establish trust than to lose trust. Therefore, similarity is calculated as

(4) Simt(P,Q)  ={Simt−1(P,Q)+(τ×(1−Simt−1(P,Q))),where  Distt(P,Q)<rSimt−1(P,Q)−(ρ×Simt−1(P,Q)),where  Distt(P,Q)≥r,

where 0 < *τ* < *ρ* < 1 and 0 ≤ Sim_*t*_ (*P*, *Q*) ≤ 1. We assume that Dist_*t*_ (*P*, *Q*) is 0 when there are no common friends between the trustor and trustee, and the initial similarity is 0.5 as the median of trust.

Upon measuring the distance, the trustor requests the information regarding the trustee from his friends who have had previous experience with the third party. We use an average value of the product of the third party's direct trust and similarity to determine the indirect trust. Indirect trust is calculated as

(5) $$\begin{array}{c} \text{I}{\text{T}}_{t}\left(P,Q\right)=\frac{\sum_{i\in \text{adj}(P)}\text{D}{\text{T}}_{t}\left(i,Q\right)\times \text{Si}{\text{m}}_{t}\left(i,Q\right)}{\left|\;i\;\right|}, \end{array}$$

where *i* ∈ adj(*P*) implies that the third party is adjacent to the trustor (*P*) and has past experiences with the trustee (*Q*).

#### 3.2.3. Combination of Direct and Indirect Trust Values for Recent Trust (RT)

Recent trust (RT) is computed using a linear combination between the direct trust value and indirect trust value at the *t*th transaction according to formulas (2) and (5). We define the recent trust of *P* to *Q* as

(6) $$\begin{array}{c} \text{R}{\text{T}}_{t}\left(P,Q\right)=\beta \times \text{D}{\text{T}}_{t}\left(P,Q\right)+\left(1-\beta \right)\times \text{I}{\text{T}}_{t}\left(P,Q\right), \end{array}$$

where *β* is the influence rate of the direct trust and 0 ≤ *β* ≤ 1. In lieu of an appropriate proportion of direct and indirect trust, we use the *β* coefficient that guarantees the minimum reflection ratio of direct trust (*v*). This is adjusted according to the transaction rate between the trustor and trustee as

(7) $$\begin{array}{c} \beta =\{\begin{array}{cc} v, & \\ \;\text{where}\;\;\beta <v & \\ v+\frac{\sum \text{number}\;\;\text{of}\;\;\text{transactions}\;\left(P,Q\right)}{\sum_{s\in S}\text{number}\;\;\text{of}\;\;\text{transactions}\;\left(s,Q\right)}, & \\ \;\text{where}\;\;\beta \geq v, & \end{array} \end{array}$$

where *S* is a set of people who have previous experience with the trustee.

#### 3.2.4. Calculation for Dynamic Trust

As mentioned previously, the trust value changes and evolves with previous experience. Based on formula (6), dynamic trust can be calculated as

(8) $$\begin{array}{c} \text{Dynamic}\;\;\text{Trust}=\left(1-\theta \right)\times \text{R}{\text{T}}_{t}\left(P,Q\right)+\theta \times \text{R}{\text{T}}_{t-1}\left(P,Q\right), \end{array}$$

where *θ* is the reflection rate of the previous trust value.

## 4. Proposed Dynamic Trust-Based Access Control Model

### 4.1. Application of Dynamic Trust Value to User's Operation

The proposed model provides a method to apply a dynamic trust value to a user operation. We assume the possible user operations in the OSN environment as shown in Table 4. The higher the access-permission level number assigned by the data owner is, the more the requestor can perform operations in the OSN. For example, a user with access permission level 4 can also perform the operations of levels 1, 2, and 3. Moreover, the access permission level is assigned and can be managed by trust level. In this manner, a trustor can control a trustee's access in this way.

### 4.2. Architecture of Dynamic Trust Access Control Model

**Figure 1:**
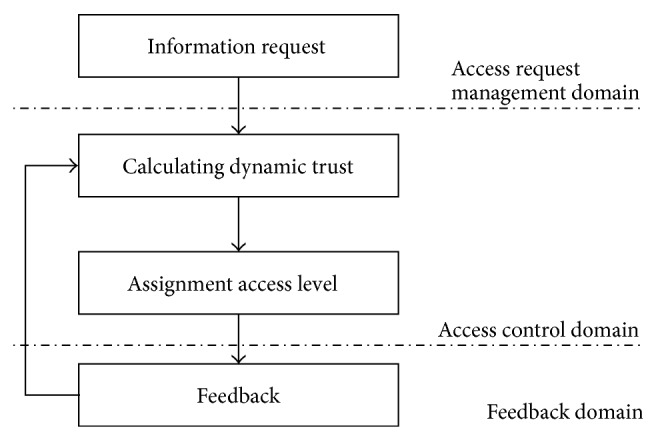
Proposed dynamic access control model.

Figure 1 shows the architecture for dynamic trust-based access control. This is categorized by access request management domain, access control domain, and feedback domain. The access request management domain has the role of scheduling the trustee's access management. It limits other operations when the trustee requests access permission. Thus, it guarantees the provision of a static environment in the dynamic OSN environment for correct trust calculation. The access control domain calculates the dynamic trust as proposed in Section 3 and assigns the access level based on the result of the calculation. The feedback domain has the role of applying the trustee's behavior to update the dynamic trust.

## 5. Experiments

### 5.1. Initial Settings

To verify the validity of the trust-based access control model, a simulation was developed using Java 1.7 SDK and a weighted digraph package. In the experiment, we set the initial variables of the trust computation as shown in Table 5. We utilized a small network with 250 nodes and 1,273 edges provided by Princeton University [18, 19].

### 5.2. Experiment 1: Changing Trust Values in Ideal Actions

**Figure 2:**
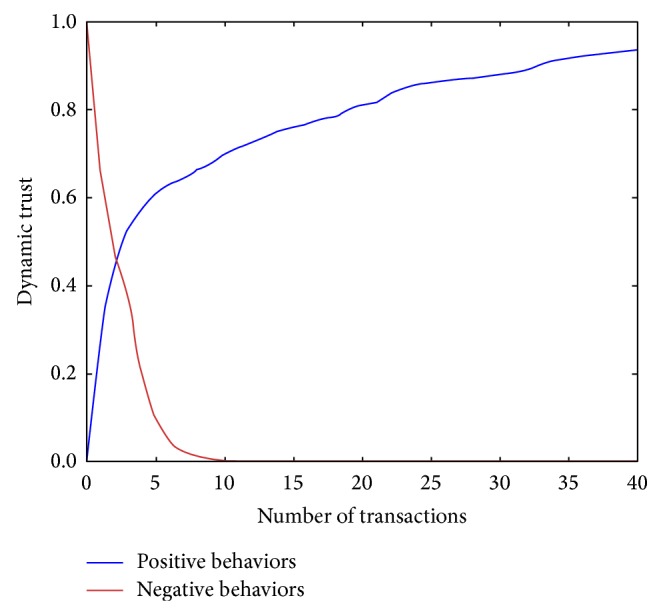
Dynamic trust by transactions.

We assumed two ideal scenarios, where the users perform continuous satisfactory actions (positive behavior) and unsatisfactory actions (negative behavior). We selected two random users in the experiment and observed the users' trust changes.  Figure 2 shows the results of the trust calculation in both cases. We set one initial user's (positive behavior) trust to zero because trust must be accumulated as we mentioned in Sections 2 and 3. Moreover, it is difficult for a user to trust others with whom the user does not have experience. However, the trust gradually increased as the trustee continued to exhibit positive behaviors. Conversely, we set the other user's (negative behavior) initial trust to one. This demonstrated that negative behavior caused the trust to decrease markedly. The experimental results reflected that trust was built slowly although it declined quickly.

### 5.3. Experiment 2: User's Strategic Actions and Mapping Trust into Operations

**Figure 3:**
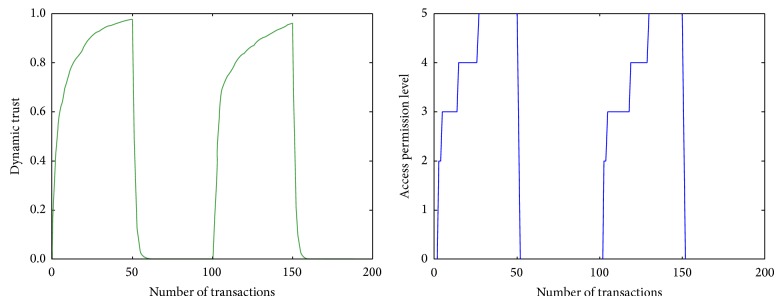
Changing of dynamic trust and access permission level against a strategic user.

We assumed that a user could choose strategic behavior and change his position on every fiftieth transaction which is from zero to fifty transaction and negative satisfaction for the subsequent fifty transactions. We assumed that maximum and minimum scores limit the fiftieth transactions because a malicious user would be focused on permanent behaviors toward the target user in the short period [15]. The dynamic change of the trust against a strategic user is represented in Figure 3. We mapped the result of the trust calculation into the access permission level. This indicates that the user's satisfactory behavior caused the access permission level to increase and to thus expand the range of available operations in a user's domain. For example, performing sustained satisfactory behaviors leads to sharing other information. Conversely, malicious or unsatisfactory behavior decreases the access permission level and reduces access to the owner's contents. For example, malicious behavior such as indiscriminate information leaking would be detected and protected easily, even though the data requestor is a trusted friend.

### 5.4. Experiment 3: Result of Simulation of the Conditions of OSN Threats

**Figure 4:**
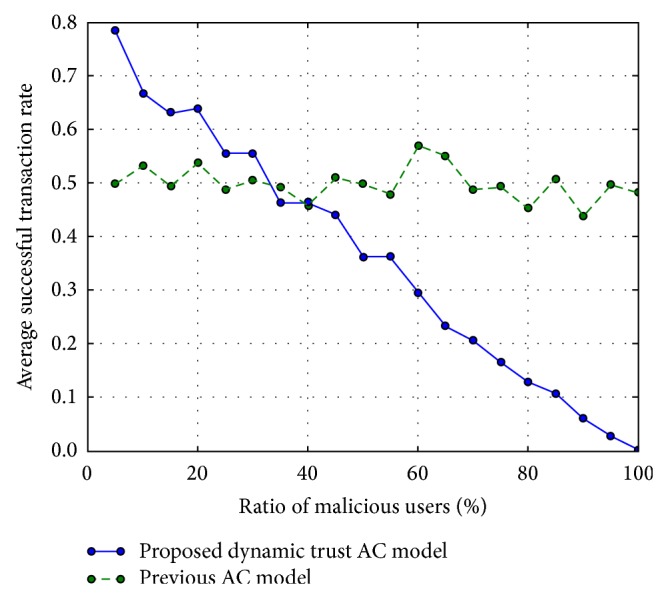
Average successful transaction rate according to ratios of malicious users.

We can observe representative threats such as the Sybil attack, malicious collectives, and malicious pretrusted users as mentioned in Section 2. Malicious pretrusted peers can select positive or negative behavior at user's choice. Further, the Sybil attack consists of multiple users who have the same identities and behavioral tendencies and malicious collectives conduct negative behavior as a group. Thus, we assumed the threat conditions and simulated those conditions every ten times, as follows. We divided all users into two groups: a virtue group of nonmalicious users and a vice group of malicious users. Each group conducted random behavior according to its trust level. For example, a user in the malicious group was assigned a low trust level initially and had more opportunities to behave badly in a trustor's domain.  Figure 4 shows the rate of the average successful transactions about the probability of a trustor giving the access permission to a trustee on average. We determined that the proposed model controlled the access permission properly compared to the previous access control models [11–14]. The traditional access control models could only filter malicious transactions when the trust level of a trustee was lower than assigned. However, the proposed model reflected the trust history of the trustee and the trust value could be changed by previous behavior. Thus, as the number of malicious users in a group increased, the rate of successful transaction became lower.

## 6. Conclusion and Future Work

This paper presented a dynamic trust calculation and its application to the access permission level that defines the range of a user's OSN access. OSN is a dynamic network environment and OSN components such as role, contents, and profile are always changing. Trust can be used as a tool to overcome the limitation of the static traditional access control mechanisms currently adopted by the major social network services such as Facebook and Linked-In. We demonstrated that the proposed dynamic trust-based access control model enables a data owner to control and protect against irresponsible or malicious information leaking. Moreover, the proposed model reduced the complexity of managing access decisions by updating trust automatically in the dynamic OSN environment.

As a future extension of the proposed access control model, real case experiments will be conducted to build more complex examples of the calculation and develop the current access control model and implementation for the platform interoperating of online social network services.

## Figures and Tables

**Table 1:** Threats related to trust in OSN.

Case	Threats
Malicious users and collectives	Users or groups who deliberately provide false information with malicious intent

Sybil attack	Users or groups who create many false accounts and fabricate the reputation of another user or group

Malicious pretrusted peers	User who adopts a negative position against the trustor after establishing reliable relationship with him

**Table 2:** Comparison of proposed dynamic trust-based access control model with previous access control models in OSN.

Categories	Contents	Previous access control model			Proposed model
		[11, 12]	[13]	[14]	
Criteria of computing trust	How to measure owner's trust against data requestor	○	—	—	○

Multiparty relationship	Relative roles of owner to control access permission	○	○	○	○

Similarity	Relative degree of other's trust similar to owner's trust because trust is subjective and transitive	—		—	○

Third party's recommendation	Third party's trust based on previous experiences with the requestor	—	○	○	○

**Table 3:** Proposed trust evaluation factors and OSN services.

Entities	Evaluation factor		Contents	User services in OSN	
				Positive	Negative
Trustor		Tendency (ta: trust ability, ts: trust sustainability, and tr: trust relationship)	A trait that leads to a generalized expectation to trust others	—	
	Internal factor	Ability (*A*)	Characteristics that enable a user to have an influence within some specific domain. The trustee may be highly competent in the owner's domain affording that person trust on tasks related to some area.	Like, link, and share	Dislike
Trustee		Sustainability (*S*)	The trustee's perception that adheres to want to access someone's domain continuously.	Hits, comment, reply, and chat	Misuse report
		Relationship (*R*)	A specific association between two or more people that binds a strong or weak tie.	Friendship, number of common relationships	Block relationship
Third party	External factor	Recommendation	The degree of third party's trust derived from previous experiences with trustee	Trust value from third party	

**Table 4:** Operation examples for user actions with trust level in OSN environment.

Access permission level	Operations	Contents of possible threats in OSN	Trust value
0	*⌀*	None	0
1	{read}	Passive recognition of information	(0–0.4)
2	{like, dislike}	Passive behavior against information	[0.4–0.6)
3	{comment}	Malicious reaction against information	[0.6–0.8)
4	{post contents, tag}	Active malicious action in owner's domain	[0.8-0.9)
5	{share}	Active information leaking	[0.9-1)

**Table 5:** Input variable for experiments.

Symbol	Contents	Value
*α*	Reflection rate of current satisfaction	**0.25**
*r*	Threshold of average distance	**0.25**
*ρ*	Punishment coefficient	**0.06**
*τ*	Reward coefficient	**0.03**
*υ*	Reflection rate of minimum direct trust	**0.25**
*θ*	Reflection rate of previous trust	**0.5**
